# Tandem machine learning for the identification of genes regulated by transcription factors

**DOI:** 10.1186/1471-2105-6-204

**Published:** 2005-08-22

**Authors:** Deendayal Dinakarpandian, Venetia Raheja, Saumil Mehta, Erin G Schuetz, Peter K Rogan

**Affiliations:** 1 School of Computing and Engineering, University Of Missouri-Kansas City, Kansas City, Missouri, USA; 2 Pharmaceutical Sciences, St. Jude's Children's Research Hospital, Memphis, Tennessee, USA; 3 Laboratory of Human Molecular Genetics, Children's Mercy Hospital, Kansas City, Missouri, USA

## Abstract

**Background:**

The identification of promoter regions that are regulated by a given transcription factor has traditionally relied upon the identification and distributions of binding sites recognized by the factor. In this study, we have developed a tandem machine learning approach for the identification of regulatory target genes based on these parameters and on the corresponding binding site information contents that measure the affinities of the factor for these cognate elements.

**Results:**

This method has been validated using models of DNA binding sites recognized by the xenobiotic-sensitive nuclear receptor, PXR/RXRα, for target genes within the human genome. An information theory-based weight matrix was first derived and refined from known PXR/RXRα binding sites. The promoter region of candidate genes was scanned with the weight matrix. A novel information density-based clustering algorithm was then used to identify clusters of information rich sites. Finally, transformed data representing metrics of location, strength and clustering of binding sites were used for classification of promoter regions using an ensemble approach involving neural networks, decision trees and Naïve Bayesian classification. The method was evaluated on a set of 24 known target genes and 288 genes known not to be regulated by PXR/RXRα. We report an average accuracy (proportion of correctly classified promoter regions) of 71%, sensitivity of 73%, and specificity of 70%, based on multiple cross-validation and the leave-one-out strategy. The performance on a test set of 13 genes showed that 10 were correctly classified.

**Conclusion:**

We have developed a machine learning approach for the successful detection of gene targets for transcription factors with high accuracy. The method has been validated for the transcription factor PXR/RXRα and has the potential to be extended to other transcription factors.

## Background

Nucleic acid binding sites recognized by transcription factors are comprised of families of short, related, often degenerate sequences that share a common function. This degeneracy may be represented in the form of a position-specific weight matrix (PWM) [1,2]. In fact, PWMs have been widely applied [2,3] and several databases host them [4,5].

Using information theory, the degree of conservation of an individual member (both known and predicted) of that family and its corresponding weight matrix may be quantified in terms of bits of information [1]. The strength of experimental binding has been shown to correlate with the predicted value of binding strength in bits for individual transcription factor binding sites [6]. We have used this approach, for example, to successfully find potential splice sites and to predict the probability that a given splice site will be used [7,8].

Regions upstream of transcription initiation sites typically contain multiple potential heterogeneous binding sites recognized by the same transcription factor. However, such sites can be found in the promoter regions of both genes that are, and those that aren't, regulated by the same factor [9], suggesting that additional sequence or structural properties are needed to discriminate true target genes from those which are not regulated by a particular factor. This paper addresses the problem of the identification of target genes for xenobiotic-sensitive transcription factors in the human genome by using a combination in tandem of information theory, a novel information density-based clustering (IDBC) algorithm and machine learning approaches for classification.

As proof of concept, binding sites recognized by the nuclear receptor transcription factor, the pregnane X receptor (PXR), are used to develop an algorithm that identify genomic target genes regulated by this factor. The algorithm exploits a PWM that accurately models the affinity of the protein for these sequences [6]. PXR is a ligand-activated transcription factor that heterodimerizes with the 9-*cis *retinoic acid receptor X (RXR) to form PXR/RXRα. Following exposure to xenobiotics like rifampin, clotrimazole, ritonavir, phenobarbital and hyperforin, and endogenous compounds like lithocholic acid steroids [10], PXR/RXRα binds response elements in the promoters of regulated genes to induce gene expression.

## Results

**Figure 1:**
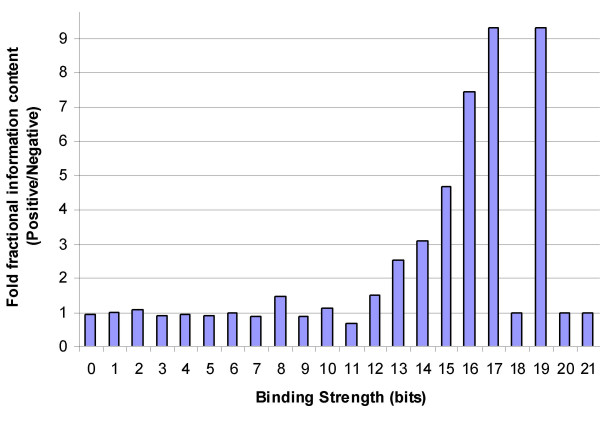
**Higher proportion of information content lies within stronger sites in promoter regions of regulated genes**. A histogram representing strengths of putative binding sites for the transcription factor RXR/RXRα is shown. The x-axis represents the binding strength of a site in bits. The y-axis represents the relative ratio between the proportions of total information found at the corresponding strength in regulated and unregulated promoter regions (10 kb upstream of respective genes).

An information theoretic approach has high recall in identifying promoter elements bound by PXR/RXRα. Given a representative weight matrix, sensitivity is essentially 100%. However, information theory weight matrices are not sufficient to discriminate between true and false positives, since binding sites were also found within the promoter regions of genes known not to be regulated by PXR/RXRα. The analysis of total information content in positive versus negative sites shows that, on average, the information is concentrated more in stronger sites in the case of regulated genes (Fig. 1). Figure 1 shows the ratios between the fractions of the sum of the information content found in regulated versus unregulated genes at each binding strength. For example, if we consider sites that have a binding strength of 17 bits, we first count the number of such sites found in regulated genes and multiply this by 17. The resulting number is then divided by the total number of bits for all sites, irrespective of strength, and this yields the average fraction of information content in a promoter region that is found in sites with a bit strength of 17. If the same calculation is carried out for genes known to be unrelated to PXR/RXRα, the ratio of the fraction computed for regulated genes to that for unrelated/unregulated genes gives the corresponding y-axis value in Fig. 1. Thus, the graph shows that a 10-fold higher proportion of total information content is found within sites having a strength of 17 bits in regulated versus unregulated promoters. However, note that this is an observation based on an average quantity, and no significant differences are evident at sites that are 18, 20 or 21 bits in strength. It was apparent from our preliminary studies (of *CYP3A4*, *CYP3A7 *and other genes) that multiple binding sites were necessary for transcriptional activation. None of the promoters examined to date contain a single strong binding site, rather they contain multiple sites. Putatively, a few moderately strong sites might increase the odds of a transcription factor recognizing a promoter that is then bound to the strongest site(s) in the region. Therefore, in this paper, we have outlined a tandem machine-learning approach for the characterization of PXR/RXRα genomic signatures that takes into account the density, strength, and location of the sites predicted by information theory. In effect, the unknown function for successful regulation of a gene that we are trying to approximate may be expressed as *F*(B, D, S) where B = the frequency distribution of binding site strengths, D = the set of distances of binding sites from the transcription start site and S = the spacing (or density-based clusters) between the sites for a given promoter region.

The information gain ratio with respect to classification of a region as positive or negative was computed for each attribute (see *Feature Selection *under Methods). The gain ratio rather than information gain was used to accommodate the fact that the number of possible states for the distribution differs from attribute to attribute. The attributes with the highest information gain ratios were considered for further analysis. Based on this analysis, the attributes with the highest gain ratios were found to be the number of clusters (0.79), total information (0.75), number of sites (0.7), information within clusters (0.67), information in top three clusters (0.62), and information over (x = 6) bits (0.5). Hence, these attributes were used for analysis with classification algorithms.

The results of the learning from the classification algorithms are summarized in Tables 1, 2. We expected to get progressively better performance in the sequence Naïve Bayesian, Decision trees, Neural Networks (Log-Sigmoid) and Neural Networks (Radial Basis Function). This is in keeping with the limiting assumption of Naïve Bayes learning that all attributes have independent probability distributions and the progressively flexible allowance for boundaries in dimensional hyperspace. For example, decision trees are limited to the use of hyperplanes that are perpendicular to some attribute or variable. Neural networks based on backpropagation relax this requirement to allow hyperplanes to adopt any orientation. The use of radial basis functions allows further flexibility in the partitioning of hyperspace. Contrary to expectation, all these methods exhibited comparable average performance. However, the NNs had the most consistent performance, i.e., they showed the least variance in performance with respect to choice of training set.

**Table 1:** Results of cross-validation by Decision Trees and the Naïve Bayes Classifier

**Method/Performance**	**DECISION TREES**	**NAïVE BAYES CLASSIFIER**
**3-way CROSS VALIDATION**		
ACCURACY	71	72
SENSITIVITY	63	83
SPECIFICITY	71	70
**LEAVE-ONE-OUT STRATEGY**		
ACCURACY	63	68
SENSITIVITY	70	79
SPECIFICITY	63	68

**Table 2:** Results of cross-validation by Neural Networks

**Method/Performance**	**NEURAL NETWORKS**	**NEURAL NETWORKS**
	**Log Sigmoid**	**Radial Basis Function**

**3-way CROSS VALIDATION**		
ACCURACY	62	73
SENSITIVITY	71	77
SPECIFICITY	62	73
**LEAVE-ONE-OUT STRATEGY**		
ACCURACY	77	78
SENSITIVITY	75	67
SPECIFICITY	77	78

We also tested the performance of the prediction methods on a test set of 13 genes (Table 3) that had not been included in either training or validation sets. The methods individually classified between 8 and 11 of the 13 genes correctly and a jury prediction correctly classified 10 of the 13 genes. The proportion of correct classification (77%) on this test set is in line with the performance noted in cross-validation of the training data (Tables 1, 2).

**Table 3:** Predictive performance on a test set of genes

***Test Gene***	***Neural Network***	***Naïve Bayes***	***Decision Tree***	***True Class***
***CYP3A4***	P	P	P	P
***CYP3A7***	N	P	P	P
***CYP3A5***	N	N	N	P
***SRP***	P	P	P	P
***CYP51A1***	N	N	N	P
***CRYZ***	P	P	P	P
***SMN2***	P	P	P	P
***HOXA9***	N	N	N	N
***CDC2L5***	N	N	N	N
***AKAP9***	P	P	N	N
***VIK***	N	N	N	N
***ATP5J2***	P	N	N	N
***PFKB4***	N	N	N	N

**Figure 2:**
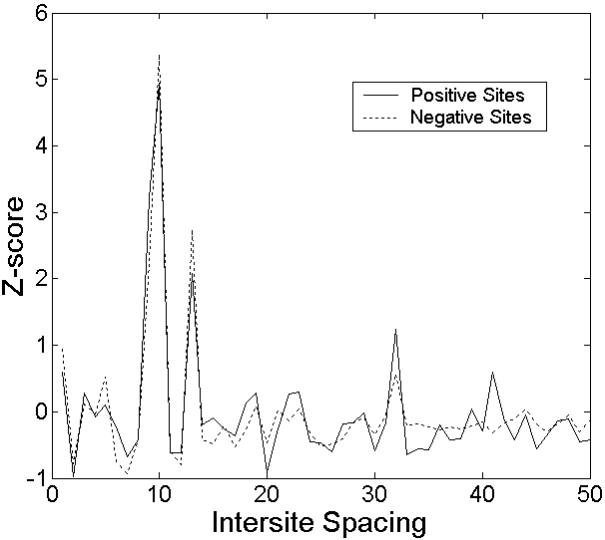
**Plot of inter-site distance and information content**. The x-axis represents the spacing between a pair of sites expressed in number of bases, whereas the y-axis represents the corresponding pair-wise sum of information for all occurrences at a given spacing. The y-axis value is expressed in terms of a Z-score – units of standard deviation from the mean. The solid line represents the curve for a set of genes known to be regulated by PXR/RXRα, while the dotted line represents genes known to be unaffected by PXR/RXRα.

One of the questions raised by the IDBC analysis was to what extent did the genomic organization of predicted binding sites determine whether a particular gene was classified as a target for PXR/RXRα. We first considered the possibility that treating DNA strands separately might for promoter regulation, however experimental studies have shown that functional PXR/RXRα sites can occur on either strand and enhance transcription of genes regulated by this factor [6]. We then looked for trends in inter-site spacing. There is a bias (Fig. 2) in the periodicity of the separations of sites within clusters, indicating a preference for separation of sites by helical turn length, ie. 10 bp (and multiples thereof), which is consistent with multimeric protein recognition across the same face of the helix. The information maxima within PXR/RXRα binding sites are also separated by 10 bp, also consistent with major groove recognition [11]. However, the length of the binding site is considerably longer than this spacing, suggesting that binding site cluster recognition may in some way be mediated by interactions involving overlapping or alternating half sites. Surprisingly, this distribution is evident in promoter regions of both genes that are regulated PXR/RXRα (solid lines, Fig 2) and those whose expression is unchanged in response to rifampin (dotted lines, Fig. 2). There was no evidence of higher order chromatin accessibility to binding sites, since there was no preference for nucleosome phasing (160–200 bp) of binding sites (not shown).

## Discussion

The importance of clustered binding sites as an indicator of a regulatory region has been noted in several studies. One of the first [12] modeled the occurrence of clusters as a Poisson process in order estimate a p-value. However, this study required exact matches to consensus sequences, rather than PWMs and did not identify putative binding sites. The objective was to maximize specificity at the cost of recall. Berman et al. [13] used a program called *CIS-ANALYST *for the recognition of clusters of binding sites in the Drosophila genome. *CIS-ANALYST *uses a window-based approach to cluster sites solely based on physical location, without regard to strength of the sites. Further, cluster boundaries are coarse because they depend on the simple rule of collapsing overlapping windows. Therefore, the size of the cluster is on the order of a multiple of the parametric window size. MSCAN [14] aims to detect regulatory regions in genomes by clustering binding sites for all transcription factors. Both CIS-ANALYST and MSCAN use PWMs [1,2], clearly a superior alternative to consensus sequence detection of binding sites. However, a fixed window size is used for the detection of clusters by MSCAN, and the computed p-values represent an upper bound. Several other studies also use a fixed-size window [15,16]. Cluster-Buster [17] scans whole genomes with PWMs using dynamic programming to efficiently compute the log likelihood ratio of a clustered model to that of a random background model. It is not clear what threshold should be used to determine if the results obtained are significant.

The algorithm, the underlying information PWMs, and the nature of the present study distinguish the current approach from previous work. IDBC uses the metric of *information density *for delineating clusters of binding sites. Thus, the criterion for clustering is not the distances separating the binding sites *per se*, but is proportional to the number of bits of information. This implies that the size of a cluster may be highly variable, being influenced by both binding strength and the number of constituent sites. Neither is a rigid requirement for the number of sites imposed (a single site could also potentially constitute a cluster), nor is the boundary of a cluster constrained. Some studies [14,17] claim to obviate the need for training data. This is strictly not true as a PWM implicitly represents a trained model, but such studies do offer the advantage of not needing a known list of regulated genes. Such methods may be valuable as preliminary screening tools, especially when there is paucity of training data. But approaches that use training data have the potential to yield higher specificity and sensitivity such as that reported in the present study. Better discrimination between regulated and unregulated genes can be achieved by having suitable positive and negative examples from which to automate learning. This will help to map a transcription factor to a comprehensive set of cognate gene targets.

Despite the high level of performance we report, it is necessary to consider why our accuracy is limited. Possible explanations are:

i) The effect of 3D higher order structure of DNA has not been taken into account in the study, other than looking for periodicity in location of the sites. This might result in a difference to accessibility of different promoter regions by transcription factors.

ii) For some of the genes, the concomitant presence of binding sites for other transcription factors might be important. Or, in general, other additional factors may be important. In other words, PXR/RXRα might be necessary, but not sufficient, for activation/repression of some of the target genes.

iii) The negative training set may be confounded by cryptic PXR/RXRα target genes whose expression did not change in response to rifampin treatment in the HepG2 hepatic cell line. Since PXR/RXRα appears to be more broadly expressed [18], it is quite plausible that some target genes containing binding site signatures may not have been activated or repressed in the HepG2 background. These false negative assignments may be rectified by analysis of expression in appropriate tissues treated with PXR ligands.

iv) The analysis has been limited to the 10 kb region upstream of each gene. Though this is likely to be the most representative region for the vast majority of genes, this may ignore the presence of control elements in other locations in a few cases.

v) The classification boundaries might be highly complex. This is supported by the fact that we noticed a slight improvement upon changing the neural network architecture from standard backpropagation with log-sigmoid functions to RBF learning. The former is theoretically limited by its use of hyperplanes while the latter uses Gaussian distributions to divide multidimensional space.

## Conclusion

We have presented a tandem machine learning approach for the computational identification of target genes for a given transcription factor. The locations and organization of binding sites alone are insufficient to discriminate genes regulated by a transcription factor from other gene targets. The strength of the approach is based on the tandem use of information-theoretic weight matrices, a novel density-based clustering approach and machine learning methods for classification. The method has been validated for the transcription factor PXR/RXRα, and has the potential for the improved identification of transcriptional regulatory targets across the entire genome [19].

## Methods

**Figure 3:**
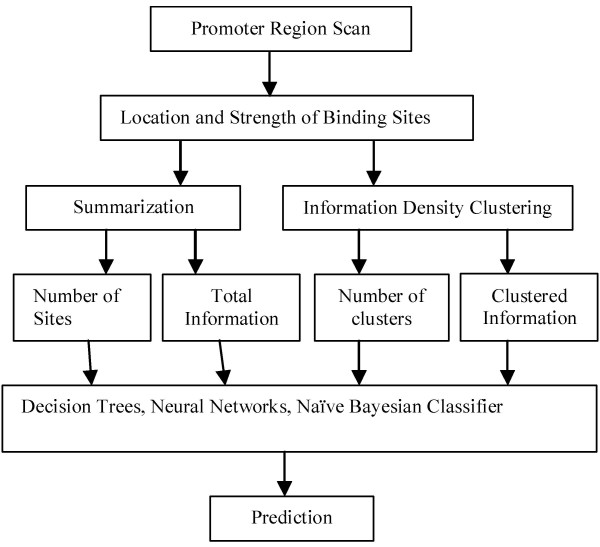
**Overview of tandem machine learning**. For each gene, the PWM representing binding sites for PXR/RXRα was used to scan the 10 kb region upstream of the transcription start site to generate a list of the location and strength of individual binding sites. This list was used to generate summary features, e.g., the total number of sites, total information content. It was also used as input for IDBC to generate clusters. A second set of summary features was extracted from the clustering obtained, e.g., total number of clusters, total information content within clusters. The combined list of features for each promoter region constituted a single data item for input to one of several machine-learning algorithm.

An overview of the general approach to the problem is given in Fig. 3. Each stage in the process was performed as follows.

### Search for potential binding sites

The information-theoretic approach for refinement of binding site models of known and predicted binding sites has been described previously [1,7,8]. The initial binding site model, derived from 15 previously reported PXR binding sites, was found to be significantly biased towards consensus sequence-like recognition sites based on their high individual information contents (R_i_) [6]. The corresponding PWM derived from this data failed to detect weak and intermediate strength binding sites and inaccurately predicted their binding affinities. With progressive model refinement incorporating newly identified, experimentally-validated sites, the R_i _values tended towards a Gaussian distribution. Using the refined PWM, the R_i _values more accurately measured the affinity of known regulatory sequences and more comprehensively identified predicted sites, consistent with the expectations from information theory [1] and the findings seen in refined models of other genome-wide protein-nucleic acid interactions [6]. This motivated our selection of the PXR/RXRα information PWM [6] for the present study.

We scanned the promoter regions (10 kb upstream of transcription start sites (TSS)) on both strands of 24 genes known to be regulated by PXR/RXRα (Table 4) for binding sites and 288 genes known to be negative based on literature and microarray data. The location and strength of binding sites for each promoter region were recorded for use in subsequent steps.

**Table 4:** Genes known to be regulated by PXR/RXRα based on [25] and/or microarray analysis

*ABCB11*	*LTB4R*
*ABCB4*	*SLC17A4*
*ABCC2*	*SLC21A14*
AV699347^1^	*SLC21A8*
*CHST7*	*SLC21A9*
*CYP2A3*	*SLC2A10*
*CYP2B6*	*UGT1A1*
*CYP2C8*	*UGT1A3*
*CYP2C9*	*UGT1A4*
*CYP3A43*	*UGT1A6*
*CYP4F3*	*UGT1A9*
*GSTA2*	*UGT2B15*

^1^This is a spliced EST, possibly an incomplete gene.

### Information density-based clustering (IDBC)

**Figure 4:**
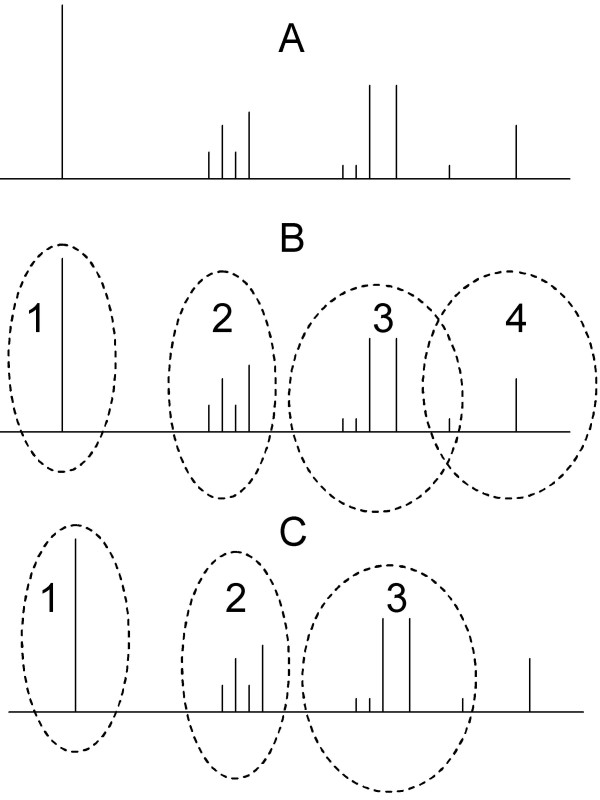
**Information Density Based Clustering (IDBC) Algorithm**. The steps of IDBC are described in the Methods section. Panel A shows the location of putative binding sites upstream of the transcription start site. The vertical height of each bar indicates the strength of the respective binding site. Panel B shows the initial list of 4 clusters derived from the first iteration of the algorithm. This includes an example of an overlap where one of the sites is shared between clusters 3 and 4. Panel C shows the result of a refining step where the overlapping point is resolved, exclusively, to cluster 3. Since the single site in cluster 4 is not strong enough to be a cluster, the final clustering has only 3 clusters.

IDBC is a modified version of the DBSCAN density-based clustering algorithm [20]. Implicitly, all clustering algorithms are based on the notion of density of data points in multi-dimensional space. Approaches such as DBSCAN explicitly use density of the distribution of data as a metric for clustering. IDBC supplements the consideration of spatial density with the additional contribution of *information density*. We define information density as being proportional to the magnitude of information content that is packed into small areas of the promoter region (see 'neighborhood information content' below). This is used as the basis for finding clusters of information rich sites. The steps of the algorithm are:

1. For each site *s*, calculate the neighborhood information content (*nic*) as being the total of pairwise sums of the information content for the site *s *and each site lying within distance *d *(number of bases) of *s*.

2. For every site *s *that has an *nic *exceeding a threshold parameter *I*, create a cluster by promoting *s *to the role of a cluster center *c *and including all sites within its neighborhood as members.

3. In the first phase of merging clusters (Fig. 4), consider all pairs of clusters with centers *c*_*i *_and *c*_*j*_. If *c*_*i *_is a member of the cluster with *c*_*j *_as its center and *vice versa*, then merge the two clusters and replace the center with the stronger of the sites *c*_*i *_and *c*_*j*_. If they are equal in strength, the center containing more sites is made the center of the new cluster, while the other center is relegated to being just a site. The process is iterated until no *c*_*i *_occurs in more than a single cluster.

4. In the second phase of merging, all *s *that belong to more than one cluster are exclusively allocated to the cluster with the stronger *c*.

5. In the re-evaluation phase, a final check is made to ensure that each cluster fulfils the criterion of minimum information density (as in step 2) after the possible reallocation of sites in the preceding step. Clusters failing the check are dissolved into individual sites.

Note that the redundant counting in the pair-wise sums of step 1 is intentional in favoring stronger sites towards becoming cluster centers. For example, given two sites *s*_*i *_and *s*_*j *_in the same neighborhood, such that *s*_*i *_has higher information content than *s*_*j*_, *s*_*i *_is more likely to qualify as a cluster center as it will have a larger *nic*.

The parameters *d *and *I *were determined as follows. A randomly chosen training sample of positive (regulated) and negative (unregulated/unrelated) genes was used for several rounds of *IDBC *with a range of values for *d *and *I*. At the end of each *IDBC *round, the total clustered information content, i.e., the sum of information content of all sites found within any cluster across the entire promoter region was computed. The final values chosen (*d *= 370; *I *= 24) were those that gave the largest difference in the mean values of total information content within clusters between the positive and negative training sets. The final clustering resulting from this algorithm allows for high total information content within clusters in more ways than one. Cluster membership can be attained by either the proximity of a few strong sites or several closely packed sites of moderate strength. Also, note that since the *IDBC *algorithm operates on the notion of information density and not an absolute inter-site distance, there is no constraint placed on a requirement for symmetric clusters. In other words, there is no enforced arbitrary bound on the location of *c*_*i *_with respect to the edge of a cluster.

### Feature selection

The following summarized features were derived as attributes for further analysis.

a) The sum of information content of all binding sites with positive R_i _in a promoter region.

b) The total number of binding sites in a promoter region.

c) Information over x bits – This is the same as "a" except that sites with R_i _less than x bits are not included in the sum. Based on training data, x was set to 6 bits.

d) The total number of clusters rich in information content found in each region by the IDBC algorithm.

e) The total information within clusters. This is the same as "a" except that sites not lying within any cluster are not included in the sum. This is a metric of how clustered the information is.

f) Information in top three clusters – The sum of information in the three highest information bearing clusters.

### Experimental data used for training and validation

Genes whose expression is regulated by PXR/RXRα (defined as "positive" in machine learning algorithms) or which are unchanged in response to treatment with the PXR ligand, rifampin, (defined as "negative") were identified by microarray analysis and from published literature. Microarray studies were carried out using HepG2-PXR cell lines were generated that stably expressed PXR. HepG2 cells were grown in MEM-alpha medium containing 10% fetal bovine serum and 1% penicillin/streptomycin at at 37C in 5% CO2. HepG2 cells were plated in P60 dishes at 50,000 cells per well. Twenty-four hours later cells were transfected with 5000 ng of human PXR-pcDNA3 (to create HepG2-PXR) or pcDNA3 (InVitrogen) (to create HepG2-NEO) by calcium phosphate co-precipitation and individual clones selected with 1000 μg/ml of G418. Clones were screened for protein expression of PXR and the clone with the highest expression of both was chosen for further study (HepG2-PXR). HepG2-PXR cells were treated with 10 uM rifampin for 48 hrs. HepG2-NEO transfected cells treated for 48 hrs with DMSO served as the control. RNA was isolated from the transfected cell lines from two independent experiments. RNA quality was verified with the "Lab-On-A-Chip" system (Agilent Technologies), reverse transcribed, and cRNA was labeled with Cy3 and and Cy5, respectively for the HepG2-PXR and HepG2-NEO lines. HG-U95 oligonucleotide microarrays (Affymetrix) were hybridized with a mixture of control and HepG2-PXR cRNA and analyzed with the Agilent Gene Array Scanner. Gene expression values were calculated using Affymetrix Microarray Suite software to compare changes in gene expression between the HepG2-NEO treated with DMSO vs. HepG2-PXR cells treated with rifampin. Genes whose expression is unchanged in response to rifampin are interpreted to be unregulated by PXR/RXRα. This assumption may only be valid for the present transfected HepG2 cell lines and it is conceivable that the regulatory status of these genes may be different in other tissues that normally express PXR/RXRα.

### Classification algorithms

The summary attributes described in the previous section were separately used as input for the construction of decision trees. Decision trees, neural networks (NN) and the naïve Bayes classifier were used separately, and as part of jury prediction. The J48 algorithm implemented in the WEKA suite of machine learning algorithms [21] based on the C4.5 decision tree builder algorithm [22] was used in this experiment.

**Figure 5:**
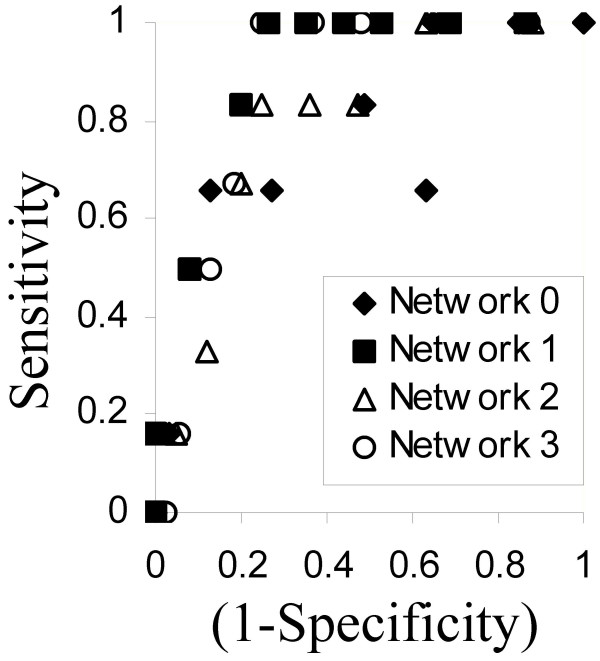
**ROC plot for Neural Network cross-validation**. The training data was divided into multiple (*n *= 4 in this figure) non-overlapping sets. Each of the *n *sets was used to train a different neural network (NN) and tested on the remaining data. A Receiver Operating Curve was generated for each trained network by calculating specificity and sensitivity for different values of the cut-off for the output value to discriminate between regulated and unregulated gene targets. The ideal curve would be collinear with the y-axis for x = 0, and then run parallel to the x-axis as the line y = 1.

The Stuttgart Neural Network Simulator (SNNS) [23] was used for neural network analysis. All attribute values were normalized by the value two standard deviations higher than the respective means observed in the training set, in order to constrain all values to lie in the range between 0 and ~1. For standard backpropagation, a single hidden layer with 2 neurons was used and the log-sigmoid function used for all layers. The latter gives a smooth output in the range of 0–1 that lends itself to probabilistic interpretation. A receiver operating curve (ROC) plot (Fig. 5) based on the training set was used to select the output value for optimal partition. Neural networks were also evaluated by using radial basis functions (RBF) [24] for the neurons in the hidden layer. In this case, the hidden layer had 3 neurons. In both cases, early stopping with keeping the size of the NN to the minimum necessary was used to reduce the possibility of overfitting.

In addition, the naïve Bayes classifier was used. The training set was used to compute a frequentist estimate of the probabilities of observing each value of the attribute in either positive or negative genes. Then, given a set of attributes for a gene in a test set, the assignment to the positive or negative class was made by considering the relative probabilities for finding such a promoter region in positive versus negative genes.

### Data-partitioning for training, validation, and testing

The data was partitioned n-ways (n = 3 or 4), with each partition being left out of the training and being validated in turn. In addition, given the small number of known positives, leave-out-one validation was also carried out, where each example was left out of the training and then subjected to prediction. Balanced training was carried out by having the same number of positive and negative examples being presented to the classification algorithms. The test set of 13 genes was not part of the training set.

The PWM was derived from 48 validated binding sites [6], including 3 individual binding sites that are present in the *CYP3A4 *and *CYP3A5 *promoter regions used to train the model. The neural network should exhibit little if any bias towards recognition of genes containing these 3 specific sequences, because it depends on multiple orthogonal features, and does not imply circularity for the following reasons: i) The sites included from *CYP3A4 *and *CYP3A5 *represent only 6% of sites in the model and less than 0.1% of those that are predicted within the respective promoter regions used to train the model. ii) The strength of these individual binding sites (see Fig. 3) is just one of the metrics used as input for the learning algorithm. Most of the training data are based on the locations of the binding sites and their spatial relationships. These criteria are unrelated to binding site strength *per se*. iii) Finally, the approach fails to yield the correct prediction for *CYP3A5*, which would had been expected should the validated binding site information contents had disproportionate effect on the NN outcome.

## Authors' contributions

DD designed the IDBC algorithm and conceived the overall machine learning strategy: feature representation and selection, choice and parameterization of machine learning algorithms, and validation of results. PKR defined the problem, suggested the use of neural networks, provided the data, the PWM, and scanned the promoter regions. SM implemented the IDBC algorithm and carried out the neural network analysis. VR experimented with other neural network architectures, performed Decision Tree and the Naïve Bayesian Classifier analyses, and carried out the cross-validation and analysis of the test set. EGS performed and provided the results of microarray expression studies. The manuscript was written by DD and PKR. Figures and tables were prepared by VR and DD.

## References

[B1] Schneider TD (1997). Information content of individual genetic sequences. J Theor Biol.

[B2] Stormo GD (2000). DNA binding sites: representation and discovery. Bioinformatics.

[B3] Altschul SF, Madden TL, Schaffer AA, Zhang J, Zhang Z, Miller W, Lipman DJ (1997). Gapped BLAST and PSI-BLAST: a new generation of protein database search programs. Nucleic Acids Res.

[B4] Matys V, Fricke E, Geffers R, Gossling E, Haubrock M, Hehl R, Hornischer K, Karas D, Kel AE, Kel-Margoulis OV, Kloos DU, Land S, Lewicki-Potapov B, Michael H, Munch R, Reuter I, Rotert S, Saxel H, Scheer M, Thiele S, Wingender E (2003). TRANSFAC: transcriptional regulation, from patterns to profiles. Nucleic Acids Res.

[B5] Marchler-Bauer A, Anderson JB, Cherukuri PF, DeWeese-Scott C, Geer LY, Gwadz M, He S, Hurwitz DI, Jackson JD, Ke Z, Lanczycki CJ, Liebert CA, Liu C, Lu F, Marchler GH, Mullokandov M, Shoemaker BA, Simonyan V, Song JS, Thiessen PA, Yamashita RA, Yin JJ, Zhang D, Bryant SH (2005). CDD: a Conserved Domain Database for protein classification. Nucleic Acids Res.

[B6] Vyhlidal CA, Rogan PK, Leeder JS (2004). Development and Refinement of Pregnane X Receptor (PXR) DNA Binding Site Model Using Information Theory: INSIGHTS INTO PXR-MEDIATED GENE REGULATION. J Biol Chem.

[B7] Rogan PK, Svojanovsky S, Leeder JS (2003). Information theory-based analysis of CYP2C19, CYP2D6 and CYP3A5 splicing mutations. Pharmacogenetics.

[B8] Nalla VK, Rogan PK (2005). Automated splicing mutation analysis by information theory. Hum Mutat.

[B9] Podvinec M, Kaufmann MR, Handschin C, Meyer UA (2002). NUBIScan, an in silico approach for prediction of nuclear receptor response elements. Mol Endocrinol.

[B10] Goodwin B, Redinbo MR, Kliewer SA (2002). Regulation of cyp3a gene transcription by the pregnane x receptor. Annu Rev Pharmacol Toxicol.

[B11] Schneider TD (1996). Reading of DNA sequence logos: prediction of major groove binding by information theory. Methods Enzymol.

[B12] Wagner A (1999). Genes regulated cooperatively by one or more transcription factors and their identification in whole eukaryotic genomes. Bioinformatics.

[B13] Berman BP, Nibu Y, Pfeiffer BD, Tomancak P, Celniker SE, Levine M, Rubin GM, Eisen MB (2002). Exploiting transcription factor binding site clustering to identify cis-regulatory modules involved in pattern formation in the Drosophila genome. Proc Natl Acad Sci U S A.

[B14] Alkema WB, Johansson O, Lagergren J, Wasserman WW (2004). MSCAN: identification of functional clusters of transcription factor binding sites. Nucleic Acids Res.

[B15] Markstein M, Markstein P, Markstein V, Levine MS (2002). Genome-wide analysis of clustered Dorsal binding sites identifies putative target genes in the Drosophila embryo. Proc Natl Acad Sci U S A.

[B16] Rebeiz M, Reeves NL, Posakony JW (2002). SCORE: a computational approach to the identification of cis-regulatory modules and target genes in whole-genome sequence data. Site clustering over random expectation. Proc Natl Acad Sci U S A.

[B17] Frith MC, Li MC, Weng Z (2003). Cluster-Buster: Finding dense clusters of motifs in DNA sequences. Nucleic Acids Res.

[B18] Lamba V, Yasuda K, Lamba JK, Assem M, Davila J, Strom S, Schuetz EG (2004). PXR (NR1I2): splice variants in human tissues, including brain, and identification of neurosteroids and nicotine as PXR activators. Toxicol Appl Pharmacol.

[B19] Gadiraju S, Vyhlidal CA, Leeder JS, Rogan PK (2003). Genome-wide prediction, display and refinement of binding sites with information theory-based models. BMC Bioinformatics.

[B20] Ester M, Kriegel HP, Sander J, Xu X (1996). A density-based algorithm for discovering clusters in large spatial databases. Proceedings of the 1996 International Conference on Knowledge Discovery and Data Mining (KDD '96).

[B21] Witten IH, Frank E (1999). Data mining: Practical machine learning tools and techniques with Java implementations.

[B22] Quinlan JR (1993). C4.5: Programs for machine learning.

[B23] Zell AKTMNST (1991). Recent Developments of the Neural Network Simulator. Proceedings of the Applications of Neural Networks Conference, SPIE.

[B24] Poggio T, Girosi F (1990). Networks for approximation and learning. Proceedings of the IEEE.

[B25] Handschin C, Meyer UA (2003). Induction of drug metabolism: the role of nuclear receptors. Pharmacol Rev.

